# Research on the evolution of biotechnology cooperation networks – a study based on patent data in China from 2004 to 2023

**DOI:** 10.3389/fpubh.2025.1437212

**Published:** 2025-03-13

**Authors:** Chongfeng Wang, Yifei Wang, Linfeng Zhong, Jie Xu

**Affiliations:** ^1^Business School, Qingdao University, Qingdao, China; ^2^School of Mathematic and Statistics, Qingdao University, Qingdao, China

**Keywords:** TERGM, biotechnology cooperation networks, networks evolution, time dependence, endogenous factors, exogenous factors, micromechanisms

## Abstract

**Introduction:**

Biotechnology has significant potential in public health, offering critical support for communicable disease control, chronic illness management, and drug development. To foster biotechnology innovation, governments increasingly incentivize cooperations among organizations, resulting in more interconnected biotechnology cooperation networks. However, research on the evolution of these networks rely primarily on static network analysis and neglect the micromechanisms under the evolution, which lead to deviations in policymaking.

**Methods:**

Using temporal exponential random graph model (TERGM), which accounts for dynamic network correlations, and based on micromechanisms framework consisting of agency, opportunity and inertia, this study analyzes the impacts of both endogenous and exogenous factors on the evolution of biotechnology cooperation networks.

**Results:**

The empirical analysis based on China’s biotechnology patent data from 2004 to 2023 reveals the following findings and policy recommendations. First, the evolution of the biotechnology cooperation networks is temporally dependent, highlighting the need for awareness of policy lags. Second, two endogenous factors – transitivity and convergence – emerge in the evolution, implying the need for government to create information platforms, establish targeted project subsidies, and enforce technical confidentiality policies. Finally, with regard to exogenous factors, the networks exhibit geographical homogeneity, implying the needs for government to promote cross-regional cooperation by establishing innovation centers and unified standards to mitigate lock-in effects and barriers.

## 1 Introduction

With the rapid advancement of technology, governments are increasingly advocating for interorganizational technical cooperations to leverage expertise from diverse organizations that can collectively drive technological innovation (1). The primary advantage of these cooperations is that they facilitate the sharing of innovative resources and enhance overall competitiveness (2). In the field of public health, effective cooperations among hospitals, universities, enterprises, and other institutions are particularly important for addressing major public health crises (3, 4). Therefore, research on the evolution of interorganizational cooperation networks in the field of public health is essential to gain a deeper understanding of its underlying principles and to provide governments with a scientific basis for formulating policies that foster its sustainable development (5).

In the public health filed, biotechnology offers efficient and accurate diagnostic tools. For example, gene sequencing technology enables the rapid identification of types of pathogens and mutations to facilitate epidemic response, and the development of molecular diagnostic technology aids in early disease detection and prevention while enhancing the level of public health prevention and control (6, 7). Furthermore, biotechnology provides new avenues for vaccine development and production through genetic engineering and cell culture. This allows for the creation of safer and more effective vaccines that serve as powerful tools against communicable diseases (8, 9). Moreover, biotechnology plays a crucial role in public health surveillance and early warning systems. Real-time monitoring systems based on advances in biotechnology can swiftly detect disease outbreaks and their spread. This information is vital for implementing timely prevention measures to curb epidemics while safeguarding public health (10, 11). It is evident that biotechnology, with its extensive applications, is highly reliant on public policies and coordinated mechanisms (12, 13). A research report from Harvard University indicates that China’s share in the global biopharmaceutical market, biopharmaceutical production capacity, and API production all rank among the world’s leaders (14). With support from the Chinese government, the biotechnology industry in China has emerged as a pivotal sector, capable of leveraging its institutional advantage to effectively deploy biotechnology in the pursuit of improving public health (15). In this study, we focus on the evolution of biotechnology cooperation networks in China.

Although current research on cooperation networks have noticed networks evolution, they have often limited their analysis to the impact of exogenous factors (16–20). As cooperations become increasingly intricate and interdependent, endogenous factors have arisen within cooperation networks. The crucial role of these endogenous factors in shaping cooperation networks has been overlooked in previous studies, which has potentially led to biased policy designs (3). Taking both endogenous and exogenous factors into consideration may offer a more comprehensive and precise perspective to guide governance in biotechnology cooperation networks and to provide valuable insights for policy-makers. The use of the exponential random graph model (ERGM) has facilitated the resolution of this issue by enabling a comprehensive consideration of both the endogenous and the exogenous factors (21). However, the ERGM is limited to cross-sectional data (22). Consequently, scholars have developed the temporal exponential random graph model (TERGM) as an extension of the ERGM to accommodate longitudinal data analysis (23, 24). By employing the TERGM, this study aims to investigate the influences of both endogenous and exogenous factors on the evolution of China’s biotechnology cooperation networks and provide insights into prospects for the biotechnology industry.

This study makes the following contributions. First, based on TERGM, we explore the influences of both endogenous and exogenous factors on the evolution of biotechnology cooperation networks as well as the time-dependent of network evolution (24). While the ERGM has significant limitations because it is applicable only to cross-sectional data, the TERGM enables longitudinal and dynamic network analysis. It therefore offers a more effective analytical method for research on technology cooperation networks. Second, this study emphasizes the micromechanisms that under the influences of endogenous and exogenous factors base on the theoretical framework proposed by Ahuja et al. (25). This framework provides a comprehensive theoretical perspective for subsequent research, addressing the theoretical shortcomings that result from the neglect of micromechanisms in previous analyses of network evolution. It also enables decision-makers to achieve a more detailed understanding and governance of the biotechnology cooperation networks. Besides, the findings of this study provide empirical support for the development of the biotechnology industry. They may also offer useful insights and guidance for other developing countries to advance their biotechnology industries, therefore contributes to the development of the global biotechnology industry.

The rest of the study is organized as follows. Section 2 provides an overview of the relevant literature. Section 3 presents the theoretical framework of the micromechanisms under cooperation networks evolution as well as the hypotheses. Section 4 introduces the methodology. In Section5, we illustrate the characteristics of cooperation networks, and the results of network evolution based on the TERGM. In Section 6, we summarize the findings and present the policy implications.

## 2 Literature review

The initial studies on the evolution of cooperation networks primarily focused on the exogenous factors and utilized the regression models. These researches can be mainly divided into two primary areas. The first area examined overall network characteristics and explores why certain features, such as small-world (26) and scale-free (27) properties, emerge in networks. The second area analyzed local connections within the network and identified factors that influence the formation of cooperations, such as accumulative advantage and homophily (28). For example, Gulati and Gargiulo (29) as well as Ahuja (30) argued that the likelihood of cooperation increases with the rise in node centrality and structural holes in the cooperation networks. Moreover, Zhao et al. (31) reported that organizations prefer partners with a greater number of existing connections. With respect to homophily, Rothaermel and Boeker (32) determined that technological complementarities and similarities among organizations influence the formation of cooperations. Yayavaram (33) reported that greater similarity in knowledge bases between two organizations increases the likelihood of cooperation. Furthermore, Zhang et al. (34) noted that geographic, cognitive, and organizational proximity exert varying effects at different stages of the cooperation networks. However, abovementioned studies based on regression models are limited by the assumption of independent, which restricts their analysis to observable and quantifiable exogenous factors.

As research progresses, the necessity to study the endogenous factors which drive networks evolution has become increasingly prominent. The emergence of social network analysis methods provides a powerful tool for studying endogenous factors. In 1996, Wasserman and Pattison introduced the ERGM to address the limitation of traditional models. This model takes the interdependence of network relationships into consideration (21) and can explore various endogenous factors which influences the formation of network edges (35). For example, Li et al. (36) reported that endogenous factors, such as “star” and “closed triangle” configurations, promote the formation of green technological innovation networks. Similarly, Ma et al. (37, 38) employed the ERGM to examine the convergence, mediation, and intermediation of organizations in technology cooperation networks. These studies demonstrate that the ERGM can compensate for the limitations of traditional regression models by incorporating endogenous factors. However, the ERGM is also constrained to analyzing static cross-sectional data and cannot account for dynamic correlations over time, which may introduce bias into the model’s fitting. To address this limitation, Hanneke et al. (23) developed the TERGM which incorporates temporal dependencies into the analysis. Specifically, the TERGM integrates discrete time-cross-sectional network data and accounts for temporal dependence between network relationships to enable a more comprehensive exploration of networks evolution while addressing the degradation issues inherent in the ERGM (24). In recent years, studies on cooperation networks have increasingly utilized the TERGM. For example, Gao and Yu (39) employed the TERGM to analyze the technological cooperation network among countries along the Belt and Road. He et al. (40) applied the TERGM to investigate the factors that influence the evolution of China’s interprovincial technological patent trade network. Shi et al. (41) employed the TERGM to examine China’s low-carbon technology cooperation network.

Based on the exploration of the exogenous or endogenous factors which drive networks evolution, scholars also propose various policy recommendations to optimize relative networks. First, transitivity is a key factor in promoting networks evolution. Zinilli et al. (42) reported that the transitivity emerges in the innovation network among Chinese cities, policymakers should focus on enhancing regional cooperation and resource integration. By setting up special funds and promoting cross-regional R&D cooperation, knowledge flow and technology diffusion can be facilitated. Chen and Wang (43) noted that with regard to the transitivity of China’s regional green technology transfer network, the network structure should be optimized to promote the rational allocation of technological resources and to avoid the excessive concentration of technology transfer. Second, the preferential attachment mechanism that arises from convergence may lead to uneven resource distribution. Liu et al. (44) noted that in the OLED technological innovation network, nodes tended to cooperate with well-connected partners, which potentially exacerbated the disadvantages for less-developed regions. To address this issue, policymakers should establish platforms to foster connections between regions, facilitate resource sharing and technology transfer. Similarly, Gao and Yu (39) noted that homophily in the Belt and Road Initiative (BRI) technological cooperation network tends to create dominance by strong players. Therefore, policymakers should promote collaborative innovation to ensure balanced cooperation. Third, the role of geographical homogeneity in improving the efficiency of collaboration has been validated by multiple studies. He et al. (40) demonstrated that geographical homogeneity reduces barriers to technology trade. Policymakers should dismantle administrative boundaries across regions, integrate technology trade platforms, and foster regional collaborative innovation. Furthermore, Shi et al. (41) emphasized that geographical homogeneity facilitates low-carbon technology cooperation. Policymakers should encourage regional collaboration and industrial clustering in low-carbon technologies. Organizational homogeneity aids cooperation but may intensify competition. Su et al. (45) reported such homogeneity emerges in the technology innovation network of the Yangtze River Delta urban agglomeration. The government should build an efficient sharing service system for technology transfer.

Abovementioned studies suggest that policymakers should optimize the structures of technological cooperation networks by fostering regional collaboration, resource sharing, and technological synergy. Moreover, policymakers should consider heterogeneity across regions and organizations by proposing tailored measures to maximize the effectiveness of technology cooperation networks. Therefore, analyzing the impacts of both endogenous and exogenous factors on the evolution of cooperation networks is of significant importance for policymaking. The biotechnology industry exhibits high levels of dynamism and rapid changes because of its inherent uncertainty, complex knowledge, and the rapid obsolescence of technological knowledge (42, 46). Given the advantages of capturing the dynamic evolution of networks, we employ TERGM to study the evolution of biotechnology cooperation networks to provide policy recommendations.

## 3 Theoretical framework and hypotheses

### 3.1 Theoretical framework

Network studies generally suggest that the evolution of networks is determined by micromechanisms. Micromechanisms is used to denote the fundamental factors that drive or shape the formation, continuation and dissolution of network connections, which manifest specifically as the pursuit by organizations of specific partners. The network micromechanisms primarily include three core components: agency, opportunity, and inertia (25). Agency implies that organizations have the motivation to actively construct network relationships. Organizations consciously choose to establish and maintain connections with other organizations to achieve their goals (47). Opportunity emphasizes that the formation of network connections often depends on specific contextual conditions. When organizations share common social backgrounds or pursue similar goals, the likelihood of establishing connections between them increases significantly (48). Inertia indicates that network relationships exhibit a degree of continuity and stability. Once a cooperative relationship is formed between organizations, accumulated cooperative norms and the mutual trust may help the relationship remain stable over time (49).

**Figure 1:**
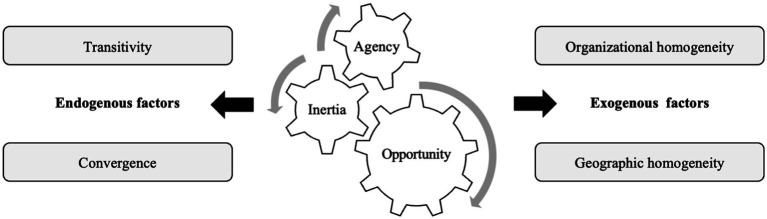
Theoretical framework of micromechanisms under the network evolution.

Based on the above theoretical framework proposed by Ahuja et al. (25) which emphasizes micromechanisms rooted in agency, opportunity, and inertia, we focus on the influences of both endogenous and exogenous factors on the evolution of biotechnology cooperation networks. Endogenous factors emphasize the influences of network structures on the evolution of the networks. These factors originate from the interactions among organizations and reflect the network’s self-adjustment. They indicate how interactions among organizations within the network drive the changes to the network. These changes rely on the network’s inherent operating logic (50). In contrast, the exogenous factors emphasize the attributes of the organizations. This type of factors, which originate from the organizations themselves, may also change the connections patterns within the network (51). The theoretical framework of this study is shown in Figure 1.

### 3.2 Influences of endogenous factors on the evolution of technology cooperation networks

**Figure 2:**
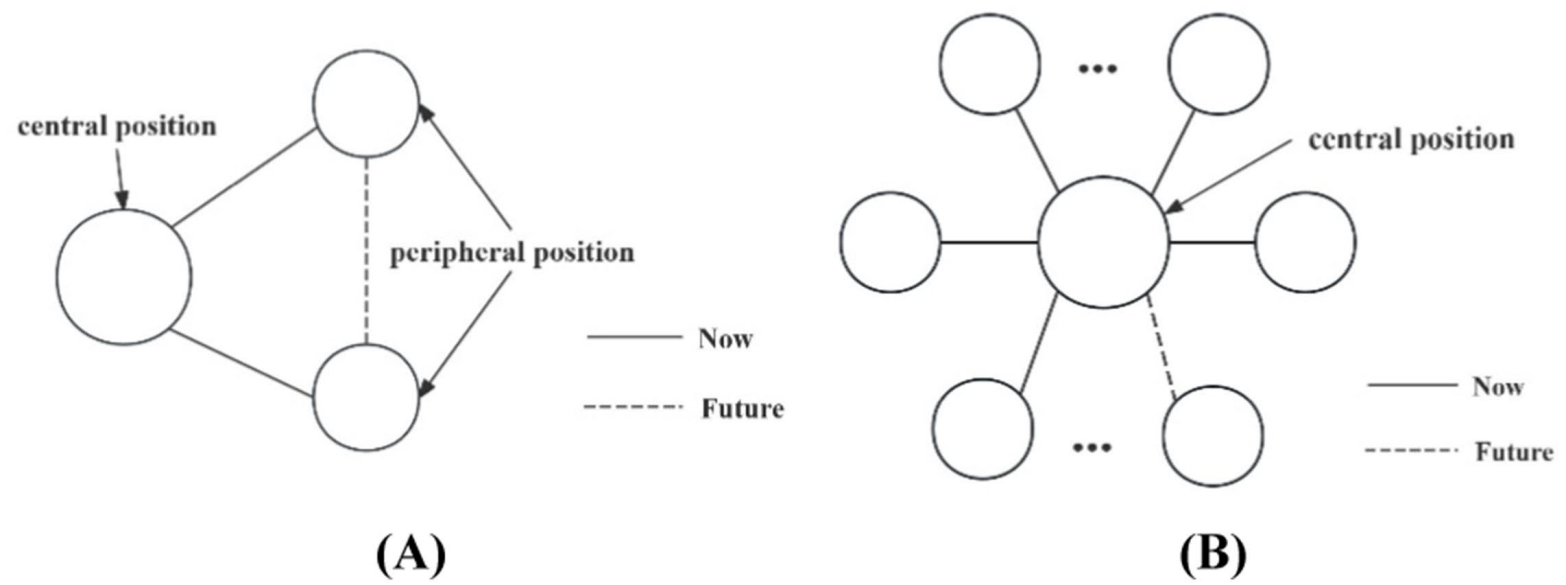
Endogenous factors. **(A)** Closed triangular structure, **(B)** Star structure.

The influences of endogenous factors can be inferred by analyzing the existing of various endogenous structures within the networks (52). Among these endogenous structures, the closed triangular structure reflects the transitivity and indicates the tendency of forming transitive edges (53). Besides, the star-shaped structure represents the convergence and reflects the tendency of organizations to cooperate with the central actors (54). These two endogenous structures have been widely used to explore the influences of endogenous factors on networks evolution (40, 55). Figure 2 illustrates examples of closed triangular structure and star-shaped structure between organizations in central and peripheral positions.

In cooperation networks, the transitivity follows the inherent logic that “a friend of a friend is a friend” (56). From the perspective of agency in the micromechanisms framework, peripheral organizations tend to form closed paths to o facilitate the efficient exchange of resources. On the one hand, these closed paths can mitigate information asymmetry when peripheral organizations interact with central organizations (57). While the information gap can hinder cooperation efficiency, closed paths limited the scope of information flow, which reduces the likelihood of information loss. On the other hand, closed paths reduce the risk of information misunderstanding when peripheral organizations rely on central organizations to convey information (58). Accurate information transmission is crucial in technology innovation. When cooperation between a peripheral organization and central organization is insufficient, other peripheral organizations could indirectly transfer the information of central organization for it, which promotes the formation of closed paths. Furthermore, central organizations also tend to encourage the construction of cooperations among peripheral organizations to prevent technology leakage (59). The formation of closed paths helps the establishing of monitoring mechanism among organizations within triangular structure, which could reduce monitoring costs (60).

From the perspective of inertia in the micromechanisms framework, the existing transitivity tend to remain stable in the networks (61). Transitivity may create a unique community structure in the networks. The behavioral norms and cooperative conventions within the community may further sustain the stable of the transitivity. Besides, the community identity could encourage organizations to maintain their affiliation with these communities, which consolidates the network’s transitivity. On the basis of above analysis, we propose the following hypothesis:

*H1*: Biotechnology cooperation networks tend to form closed triangular structures during the evolution.

In cooperation networks, the convergence is reflected in the tendency of central organizations to cooperate with more organizations, thereby expanding central organizations’ network scale and forming star-shaped structures (38, 58, 62). In cooperation networks, an organization’s number of partners increases, the likelihood that other organizations cooperate with it also rises. From the perspective of agency in the micromechanisms framework, central organizations seek to expand their network scale to increase their influences, acquire resources, and gain competencies. Furthermore, peripheral organizations are also inclined to cooperate with central organizations to access to resources which help to reduce the risk of research and development (62).

From the perspective of opportunity in the micromechanisms framework, central organizations can access more information about potential partners due to their network positions. Furthermore, central organizations are usually high visibility in the cooperation networks, which creates opportunities for peripheral organizations to see them (63, 64).

From the perspective of inertia in the micromechanisms framework, once a central organization already cooperates with many organizations and form a star-shaped structure, it may prefer to maintain this structure to keep network reputation and status (65, 66). Furthermore, peripheral organizations may also prefer to maintain star-shaped structures to gain resources through cooperation with central organizations continuously. Besides, peripheral organizations are usually reluctant to terminate cooperations with central organizations, given the latter’s significant reputation and status. In sum, we propose the following hypothesis:

*H2*: Biotechnology cooperation networks tend to form star-shaped structures during the evolution.

### 3.3 Influences of exogenous factors on the evolution of technology cooperation networks

The influences of exogenous factors can by inferred by analyzing the existing of various homogeneities within the networks. Previous studies indicate that the cooperation between organizations that share similar attributes not only benefit resources accessing but also enhance information exchanging (67, 68). Previous research had widely discussed the effects of organizational and geographical homogeneity on cooperation networks evolution (34, 69, 70).

Organizational homogeneity emphasizes the types of organizations. From the perspective of agency in the micromechanisms framework, organizations are highly motivated to seek same organizational type partners to enhance innovation capacity and market competitiveness. On one hand, organizational homogeneity usually means similar organizational structures, workflows, management styles, and research backgrounds (71). These similarities help to strengthen the knowledge absorptive capability during cooperations (72). On the other hand, organizational homogeneity could mitigate concerns about the uncertainty of cooperative organizations’ behavior, such as inadvertent knowledge leakage (33, 73).

From the perspective of inertia in the micromechanisms framework, cooperations between same type organizations tends to be stable (74). Cooperative organizations with same organizational type often have similar business models and industry standards, which help them build deep trust and shared goals, making them willing to sustain cooperation (75). On the basis of above analysis, we propose the following hypothesis:

*H3*: Organizational homogeneity contributes positively to the evolution of biotechnology cooperation networks.

Geographical homogeneity emphasizes the spatial aggregation of organizations (38). From the perspective of agency in the micromechanisms framework, organizations that cooperated in close regions could benefit from the advantages of face-to-face communication. For the easier transfer of tacit knowledge and lower communication costs accompanied by face-to-face communication, organizations may actively seek to cooperate within close regions (76, 77).

From the perspective of opportunity in the micromechanisms framework, regional embedded information could enable organizations to identify potential cooperation opportunities (78, 79). Geographical homogeneity facilitates organizational participation in industry-related activities, such as industry exhibitions, seminars, and other events. These events serve as important opportunities for organizations to obtain regional embedded information and establish cooperations within same regions (28). In sum, we propose the following hypothesis:

*H4*: Geographic homogeneity contributes positively to the evolution of biotechnology cooperation networks.

## 4 Methods

**Figure 3:**
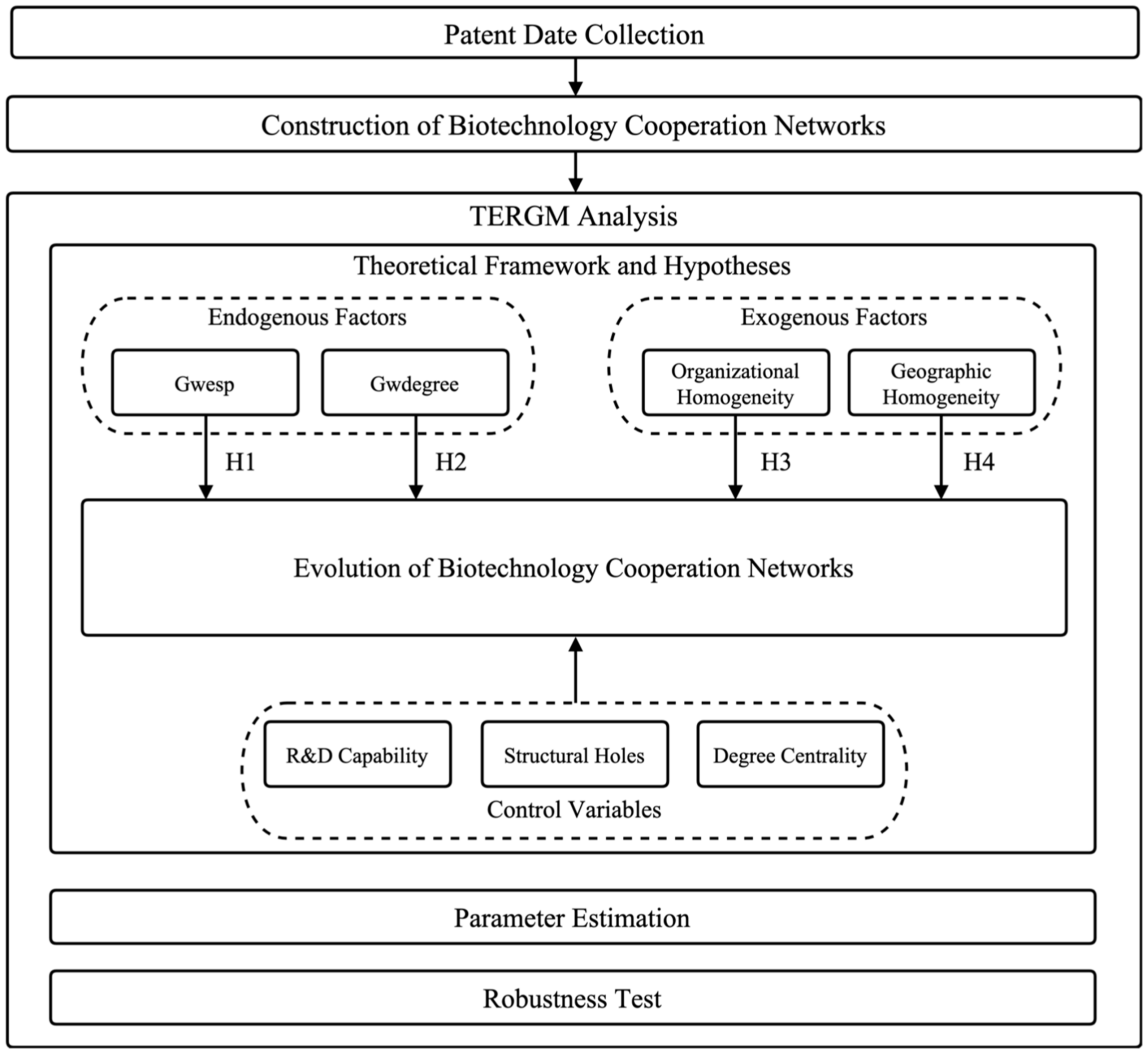
Analytical framework.

Figure 3 illustrates our analytical framework for using the TERGM to test the hypotheses proposed in this study. The major components are described following.

### 4.1 Construction of biotechnology cooperation networks

Patent-based, project-based, and academic paper-based cooperations are often used to construct networks in empirical research on technological innovation (80–82). In this study, the construction of networks through project-based cooperation and academic paper-based cooperation faces significant challenges. The contents of projects in the biotechnology industry are usually confidential, which make data unavailable in public source. Project-based cooperation data could not ensure integrity and accuracy. Although academic paper-based cooperation data are public, it cannot emphasize the entire process from technology development to industrialization (83, 84). Compared with project-based data and academic paper-based data, patent data are widely used for their usability and operability (85, 86). They not only reflect the technology innovation achievements but also possess clear legal and economic value (87). We collected China’s biotechnology patent data to construct cooperation networks in this study. In recent years, China’s biotechnology industry has grown significantly and became a key player in biotechnology industry (14, 88).

Although relying solely on China may make it difficult to capture the whole picture of international cooperation (89), this study mainly emphasizes national policy recommendations based on evolution of cooperation networks. Given that policymakers in different nations may have distinct policy objects and operate within varying policy environments, a nation-specific focus is more suitable for generating effective policy recommendations.

This study collected biotechnology patent application data of China spanning 2004 to 2023 from Patent Information Search Platform.^1^ We constructed biotechnology cooperation networks with the organization as the node and the joint patent application as the edge. The processing of patent data was performed as follows:

1. Based on the International Patent Classification (IPC) codes of the biotechnology defined by the Organization for Economic Co-operation and Development (OECD) (90), namely, A01H1/00, A01H4/00, A61K38/00, A61K39/00, A61K48/00, C02F3/34, C07G11/00, C07G13/00, C07G15/00, C07K4/00, C07K14/00, C05K16/00, C07M17/00, C00K19/00, C12M, C12N, C12P, C12Q, C12S, G01N27/327, G01N33/53*, G01N33/54*, G01N33/55*, G01N33/57*, G01N33/68, G01N33/74, G01N33/76, G01N-33/78, G01N/88, and G01N133/92, we collected patent data spanning 2004 to 2023 on January 19 in 2024, and finally obtained 425,781 records.
2. We then divided the time window and filtered the nodes. The TERGM is a statistical model that can portray the evolution of networks. It can be used to analyze network data from multiple time periods and to explore the temporal dependence of network relationships. Appropriate time intervals are usually chosen to reduce computational difficulty (22). Previous research indicates that the duration of cooperations typically range from 3 to 5 years (91). Therefore, we adopted a time interval of 5 years and divided the period of 2004–2023 into four interval segments. Subsequently, we kept organizations which had at least one cooperative partner in at least one of these segments, resulting in a sample of 760 organizations. To establish biotechnology cooperation networks and calculate network statistics, we extracted patent information to obtain organizations’ names, application dates, and country/province codes.

### 4.2 Temporal exponential random graph model

The ERGM, as a network statistical model, is mainly used to test whether networks exhibit theoretically assumed structural tendencies (21). The TERGM is an extended dynamic ERGM that further considers the dynamic changes in the networks from the previous state to the current state and captures the evolution characteristics of the networks (23). The selection of the TERGM is justified by its theoretical alignment with our research objectives and empirical compatibility with available data. The Stochastic Actor-Oriented Model (SAOM) hypothesizes that network evolution arises from continuous-time behavioral adjustments by actors, making it particularly suitable for modeling micro-level relationship dynamics (92). However, their reliance on high-frequency temporal data conflicts with our panel dataset. Besides, compared with SAOM, TERGM is more suitable to explore the influences of network structures (93). The Agent-Based Model (ABM) is good at simulating the impact of micro-level interactions on macro-level phenomena, but the higher-order dependencies it describes is somewhat opaque. For this reason, in situations where the primary goal of analysis is to evaluate specific types of higher-order network dependencies, TERGM will be more straightforward (94). Conventional time-series approaches such as the Autoregressive Integrated Moving Average (ARIMA) and the Vector Autoregression (VAR) are inherently limited by their inability to account for relational interdependencies central to network dynamics (95, 96). In contrast, TERGM provides a framework that incorporates both endogenous and exogenous factors, thereby addressing the limitations of conventional time-series approaches. By modeling network evolution as a discrete-time process driven by micromechanisms, TERGM captures how networks emerge from the interplay of individual interactions and organizational norms (24), which aligns precisely with our objective to provide the basis for policy making.

The following Equation (1) presents the typical formal representation of the ERGM:


(1) $$P_{\theta ,Y}\left(Y=y\right)=\frac{\exp\left\{\theta_{A}^{T}Z_{A}\left(y\right)\right\}}{k\left(\theta ,y\right)}$$

where Y denotes all possible networks, y denotes the observed networks, and the constant $k\left(\theta ,y\right)$ is employed to ensure that the probability of a new network structure emerging remains within the range of 0 to 1. A is the set of all network structures and node attribute statistics, $Z_{A}\left(y\right)$ represents the network statistics of A, and $\theta_{A}$ represents the parameter vector. The significance, direction (positive or negative), and magnitude of the estimated $\theta_{A}$ can determine the extent to which its corresponding node attribute or network structure statistic contributes to the connection within the networks.

The ERGM can be modified to include the k -order time dependence of the observed network y (97):


(2) $$P\left(y_{t}|y_{t-k},\ldots ,y_{t-1},\theta \right)=\frac{\exp\left\{\theta_{A}^{T}Z_{A}\left(y_{t-k},\ldots ,y_{t-1}\right)\right\}}{k\left(\theta ,y_{t-k},\ldots ,y_{t-1}\right)}$$

Equation (2) specifies the TERGM of a single network at a single moment, and the joint probability of observed the network between moments k+1 and T is Equation (3):


(3) $$P\left(y_{k+1},\ldots ,y_{T}|y_{1},\ldots ,y_{k}\right)=\overset{T}{\underset{t=k+1}{\prod }}P\left(y_{t-k},\ldots ,y_{t-1},\theta \right)$$

Scholars have suggested that the maximum pseudolikelihood estimation (MPLE) used for estimating TERGM parameters may be limited by inadequate sample randomization and inaccurate parameter confidence interval estimation (98). To address these limitations, a bootstrap-based MPLE has been proposed. Compared with the traditional method of conditioning the remaining parts after removing the network relations, this method conditions the sample data extracted using the bootstrap method. By employing the bootstrap method, more randomly sampled data can be obtained, thereby increasing the precision of the parameter interval estimation (99). Although more repetitions result in smaller simulation errors, the running time increases. Therefore, we set 1,000 repetitions in the bootstrap method (22).

### 4.3 Factors in TERGM

#### 4.3.1 Endogenous factors

We employ geometrically weighted edge sharing partners (Gwesp) and the geometrically weighted degree (Gwdegree) to represent transitivity and convergence, which are commonly used as endogenous structural variables within the TERGM (41, 42, 100). Gwesp is a function of the edgewise shared partner statistic $Esp_{k}\left(y\right)$ , defined in Equation (4):


(4) $$e^{\alpha }\overset{n-2}{\underset{k=1}{\sum }}\left\{1-{\left(1-e^{-\alpha }\right)}^{k}\right\}Esp_{k}\left(y\right)$$

where $Esp_{k}\left(y\right)$ refers to the number of edges with exactly k shared partners, and α is a decay parameter. A larger decay parameter indicates slower decay (100). Gwesp measures transitivity, which manifests as a closed triangular structure in the network and reflects the tendency of two organizations that share a partnership to also be connected within the network (101). For computational ease, the decay parameter of Gwesp is set to the default value of 0.5 (22).

Gwdegree is a function of $D_{k}\left(y\right)$ , which denotes the number of nodes in network y that have k neighbors in the network, defined in Equation (5).


(5) $$e^{\alpha }\overset{n-2}{\underset{k=1}{\sum }}\left\{1-{\left(1-e^{-\alpha }\right)}^{k}\right\}D_{k}\left(y\right)$$

Broadly, Gwdegree manifests as a star structure in the network that measures convergence and reflects the tendency of organizations to gather more partners (101). For computational ease, the decay parameter of Gwdegree is also set to the default value of 0.5 (22).

#### 4.3.2 Exogenous factors

We employ two exogenous factors, namely, organizational homogeneity and geographic homogeneity in TERGM. Organizational homogeneity refers to the tendency of organizations with same type to cooperate more frequently (38, 85, 102). Organizations with names ending in “university” or “college” were labeled as “1”; those ending in “company” or “society” were labeled as “2”; those ending in “institute” were labeled as “3”; and those ending in “hospital” were labeled as “4.” Organizations that share the same label are considered to exhibit organizational homogeneity.

Geographic proximity refers to the spatial closeness of organizations within a geospatial context. In academic research, various methods are employed to measure geographic proximity, including the physical distance measurement and geocoding matching techniques. The most straightforward method of measuring physical distance between two organizations is calculating the straight-line distance or travel distance between them (43, 103, 104). This method effectively captures the degree of spatial separation between organizations, proving particularly value in research scenarios that require precise spatial measurements. The geocoding matching method identifies geographical homogeneity among organizations by extracting their geocodes (e.g., country, province, or city codes) (37, 38, 85). This method is widely applied to evaluate geographical proximity among organizations using patent data. In this study, the geocoding matching method is utilized to extract national or provincial codes from patent information to assess geographic homogeneity. If two organizations share the same geographic code, they are considered to exhibit geographic homogeneity.

#### 4.3.3 Control variables

To address potential endogeneity issues and enhance the robustness of the TERGM analysis, this study incorporates three control variables: R&D capability, degree centrality, and structural holes. These variables have been widely acknowledged in prior studies as significant factors that influence network evolution (105–107). Controlling for these factors could mitigate biases arising from omitted variable. Specifically, R&D capability represents the technological resources and innovation potential of organizations. We counted the number of patents in each period to measure the R&D capability of each organization in different time periods (38, 105).

Degree centrality indicates organizations’ network prominence (97, 106). The Equation (6) for calculation is presented below:


(6) $$C_{ADi}=\overset{n}{\underset{j=1}{\sum }}X_{ij}\left(i\neq j\right)$$

where $C_{ADi}$ represents the degree centrality of node i ; n represents the total number of nodes; $\overset{n}{\underset{j=1}{\sum }}X_{ij}$ is the count of direct links connecting node 𝑖 with its neighboring nodes; and $X_{ij}$ is a 0–1 variable, which is 1 when node i has a connecting edge with node j and 0 otherwise ( i≠j , excluding the association of i with itself).

Structural holes quantify organizations’ brokerage capacity. With reference to Burt (107), structural holes can be measured using the constraint index. On the basis of the network data, this study used UCINET software and the following Equation (7):


(7) $$C_{ij}={\left(P_{ij}+\underset{q}{\sum }P_{iq}P_{\mathrm{qi}}\right)}^{2}\left(q\neq i,j\right)$$

where $C_{ij}$ represents the constraint index of node i by node j and $P_{ij}$ represents the percentage of time and effort that node i spends on node j . Since the biotechnology cooperation network constructed in this study is an unweighted network, the value is equal to the inverse of the number of partners of node i . The higher the constraint index is, the smaller the value of the structural holes. Therefore, $2-C_{ij}$ is used to measure the structural hole of the organizations. A specific explanation of the variables is given in Table 1.

**Table 1:** Variable description and interpretation.

Variables		Configuration	Interpretation
Endogenous factors	Edge	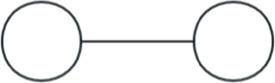	Constant term
	Gwesp	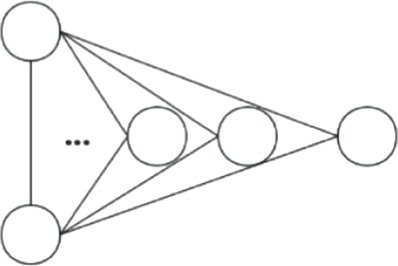	Is there a tendency to form closed triangular structures? Is there transitivity in the evolution of cooperation networks?
	Gwdegree	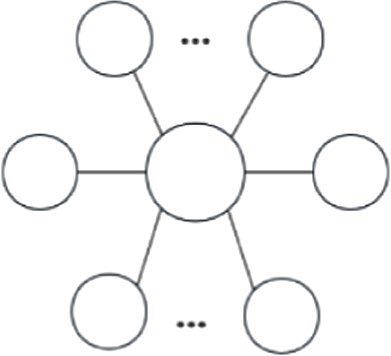	Is there a tendency to form star structures? Is there convergence in the evolution of cooperation networks?
Exogenous factors	Organizational homogeneity	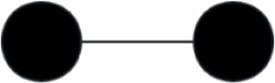	Do organizations with the same organizational type tend to cooperate with each other?
	Geographic homogeneity		Do organizations with the same geographic location tend to cooperate with each other?
Control variables	R&D capability	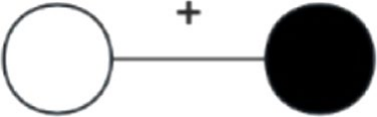	Do organizations with high R&D capability attract more cooperation?
	Degree centrality		Do organizations with high degree centrality attract more cooperation?
	Structural holes		Do organizations that occupy structural holes attract more cooperation?

## 5 Results

### 5.1 Characterization of the biotechnology cooperation networks

**Figure 4:**
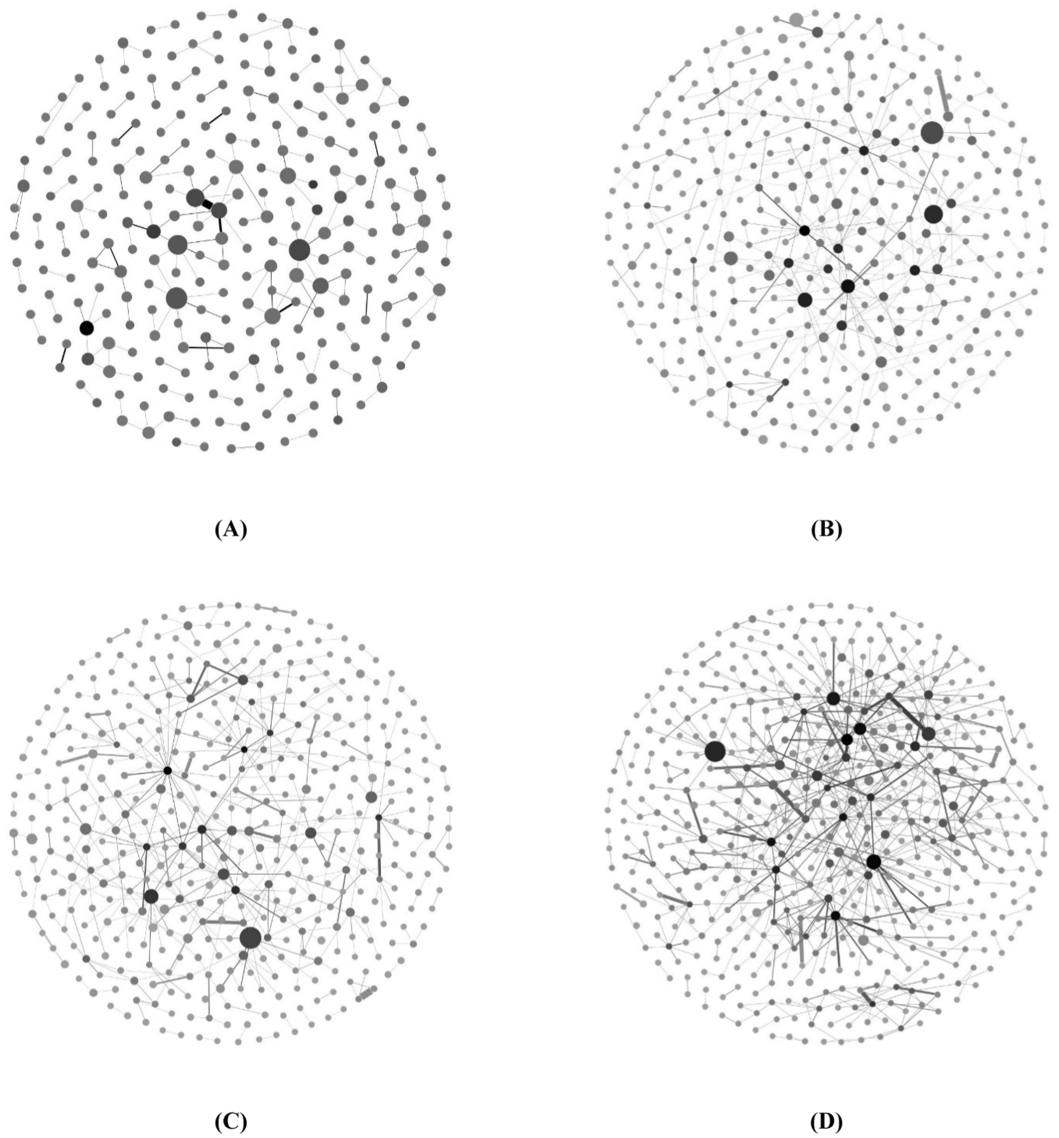
Evolution of biotechnology cooperation networks. **(A)** Cooperation network from 2004 to 2008, **(B)** Cooperation network from 2009 to 2013, **(C)** Cooperation network from 2014 to 2019, **(D)** Cooperation network from 2019 to 2023.

We used Gephi software to visualize biotechnology cooperation networks (as shown in Figure 4). The network nodes indicate organizations, and the edges of the network indicate cooperations. The size of the nodes represents the number of partners affiliated with the organizations, whereas the depth of color indicates their research capability measured by the number of patents. Additionally, the thickness of edges signifies the number of cooperations between these organizations. To visualize the cooperation networks clearly, we deleted the isolated nodes.

Although large nodes and medium-large nodes exist in cooperation networks, the number of small nodes remains significant, which indicates that the technology cooperations are not sufficient. There are fewer dark-colored nodes in the network, indicating fewer organizations with robust R&D capabilities in the cooperation network. Over time, the frequency of thicker edges in the network increases, although thin edges continue to dominate in terms of proportion. This observation suggests that within biotechnology cooperation networks, despite an overall rise in cooperation among organizations, only a few engage in more frequent cooperative endeavors. Compared with the obvious increase in network size in the first two periods, the increase in network size in the last two periods is less obvious, but the network density increases. These findings indicate that during network evolution, the expansion of biotechnology cooperation networks will eventually reach a plateau following a period of substantial growth. However, a steady increase in cooperation among organizations is anticipated.

This study employed UCINET software to examine the structural properties of the biotechnology cooperation networks. Eight indicators—network size, edges, average degree, network density, modularity, connected components, average clustering coefficient, and average path length—were measured. The detailed results are summarized in Table 2.

**Table 2:** Characteristic of biotechnology cooperation networks.

Indexes	2004 to 2008	2009 to 2013	2014 to 2019	2019 to 2023
Nodes	255	386	450	516
Edges	197	383	474	705
Average degree	0.518	1.008	1.247	1.855
Density	0.001	0.001	0.002	0.002
Modularity	0.900	0.903	0.879	0.793
Connected component	574	429	357	277
Average clustering coefficient	0.187	0.179	0.118	0.137
Average path length	4.116	7.311	6.679	4.986

According to Table 2, the network size shows a significant growth trend. It increased substantially from 255 in 2004–2008 to 516 in 2019–2023. Similarly, the number of edges increased from 197 in 2004–2008 to 705 in 2019–2023. This indicates that during the evolution, a growing number of organizations actively participate in technological cooperation, leading to a steady rise in the number of cooperative relationships. However, the average degree of organizations gradually increased to between 1 and 2, indicating that each organization, on average, established cooperations with only one or two other organizations. The network density remained relatively low (between 0.001 and 0.002), demonstrating that connections in the biotechnology cooperation network are insufficiently tight and that the level of cooperation requires further improvement. Nevertheless, given the growth trends in network size and the number of edges, an improvement in the level of cooperation can be anticipated.

Furthermore, this study examined the tightness of the network connections and the efficiency of network transmission. In terms of the tightness of connections, the average modularity is 0.869. The relatively high value in the initial period suggests that the network has a distinct modular structure characterized by relatively independent modules with strong internal connections but limited external connections. However, since 2014, modularity has declined and internal connections have increased, reducing the clarity of the modular structure. Furthermore, the number of connected components has decreased annually, indicating a continuous increase in network connectivity. At the same time, the number of isolated organizations has gradually declined with many have integrated into a larger connected component. This integration has expanded the scope and depth of cooperation. The average clustering coefficient decreased in the first three period, reflecting weak clustering among network nodes and sparse local connections. However, it rebounded to 0.137 in 2019–2023, indicating an improvement in the tightness of local connections in the later period. In terms of transmission efficiency, the average path length reflects the efficiency of information or resource transmission within the network (108). It increased significantly from 2009–2013, indicating that during this period, the connections among organizations in the biotechnology cooperation network were complex. The subsequent decrease suggests that the efficiency of information transmission improved.

Organizations with a high number of partners and collaborations with a high number of times in the four periods are shown in Tables 3, 4. As shown in Table 3, the number of partners of important organizations has increased annually. In particular, the Shanghai Institutes of Biological Sciences, Shanghai Jiao Tong University and Tsinghua University have extensive partners. As shown in Table 4, the cooperations with a high number of times mainly present between universities and research institutes. Cooperations with a high number of times between universities and enterprises remains relatively limited. Furthermore, many cooperations are among geographic homogeneous organizations, indicating that cross-regional cooperations are not yet widely realized in biotechnology cooperation networks.

**Table 3:** Representative organizations at each period.

Time	R&D organizations	No. of partners
2004 to 2008	Tsinghua University Shanghai Institutes for Biological Sciences	8
	East China University of Science and Technology	7
	Fudan University	6
2009 to 2013	Shenzhen Huada Gene Technology Co.	14
	Shanghai Jiao Tong University	12
	Sun Yat-sen University Shanghai Institutes for Life Sciences Chinese Academy of Sciences China Agricultural University	10
2014 to 2019	Shanghai Institutes for Biological Sciences	19
	Kyoto University	16
	Tsinghua University Institute of Microbiology Chinese Academy of Sciences	12
2019 to 2023	Nanjing Agricultural University	19
	Zhejiang University	18
	China Agricultural University Shanghai Jiao Tong University	17

**Table 4:** Representative cooperations at each period.

Time	Edge	No. of cooperations
2004 to 2008	Fudan University and Shanghai Human Genome Research Center	52
	Shanghai Human Genome Research Center and National institute of parasitic diseases Chinese Center for Disease Control and Prevention	16
2009 to 2013	Zhejiang University of Technology and Hangzhou Zhongmei Huadong Pharmaceutical Co.	37
	Massachusetts Institute of Technology and Society of Fellows at Harvard University	14
2014 to 2019	Novartis AG and University of Pennsylvania Board of Trustees	32
	Hebei Agricultural University and Cotton Research Institute of the Chinese Academy of Agricultural Sciences	15
2019 to 2023	South China Agricultural University and Institute of Biotechnology, Chinese Academy of Agricultural Sciences	15
	Nanjing University of Technology and Zhengzhou University	14

### 5.2 Analysis of the evolution of biotechnology cooperation networks

Drawing on the research of Wu et al. (109), we constructed different models in two stages. In the first stage, three types of ERGMs were applied to the biotechnology cooperation network from 2019 to 2023 to identify the model components of the TERGM. The parameters of each model were estimated using the Markov chain Monte Carlo maximum likelihood estimation (MCMC MLE) method in the STATNET program within R software. Model 1 focused on the endogenous factors, whereas Models 2 and 3 sequentially incorporated exogenous factors and control variables. On the basis of the Akaike information criterion (AIC) and Bayesian information criterion (BIC), Model 3 demonstrated superior explanatory power compared with Models 1 and 2. In the second stage, based on the components of Model 3, the TERGM was introduced to analyze the evolution of the biotechnology cooperation network from 2004 to 2023. The parameters of Model 4 were estimated using the bootstrap-based maximum pseudolikelihood (MPL) method implemented in the XERGM program within R software. This methodological adjustment was made to better accommodate the analysis of time series data and to accurately capture the evolution of the biotechnology cooperation network over the long term (Table 5).

**Table 5:** Estimation results of the ERGM and TERGM.

	Variables	Model 1	Model 2	Model 3	Model 4
Endogenous factors	Edges	−5.30*** (0.06)	−5.97*** (0.09)	−19.8147*** (0.9490)	−14.1762* [−15.5812; −10.8180]
	Gwesp	1.09*** (0.06)	0.71*** (0.06)	0.1323* (0.0604)	0.4248* [0.3359; 0.7946]
	Gwdegree	−1.40*** (0.06)	−1.10*** (0.10)	1.5282*** (0.2007)	0.7867* [0.7281; 1.0289]
Exogenous factors	Organizational Homogeneity		0.35*** (0.07)	0.2359** (0.0845)	0.0968 [−0.0585; 0.2461]
	Geographic homogeneity		2.12*** (0.08)	2.8340*** (0.0875)	2.6206* [2.4420; 2.7402]
Control variables	R&D capability			0.0002* (0.0001)	0.0001 [−0.0003; 0.0004]
	Degree centrality			0.0211 (0.0147)	0.0731* [0.0253; 0.2973]
	Structural holes			4.8168*** (0.3564)	3.2341* [1.2796; 3.8428]
Time-dependent effect	Stability				1.4867* [1.3784; 1.7632]
AIC BIC		9244.23	8387.15	7160.77	
		9275.95	8440.02	7245.35	

In Models 1 to 3, the standard deviations are reported in parentheses. ***, **, and * represent statistical significance at the 0.1, 1, and 5% levels, respectively. In Model 4, * represent 0 outside the confidence interval.

According to Model 4, the estimated coefficient of stability (1.4867) demonstrates a significantly positive time dependence in the formation of cooperative relationships within biotechnology cooperation networks. This finding indicates that previous cooperative relationships play a crucial role and highlights the presence of temporal dynamics in the evolution of the cooperation network. Referring to the study of Liu and Chen (110), this result also suggests that the network evolution path is more reflective of progressive development than leapfrog development. Hanneke et al.’s (23) study of time dependency showed that the TERGM has stronger explanatory power than the ERGM does. Therefore, it is reasonable to employ the TERGM to analyze the evolution of biotechnology cooperation networks.

With respect to endogenous factors, the estimated coefficient of edges is significantly negative (−14.1762), indicating low network density and an overall loose network structure. The estimated coefficient of Gwesp is significantly positive (0.4248), suggesting that biotechnology cooperation networks tend to form closed triangular structures. This implies that when multiple organizations cooperate simultaneously with a single organization, they are more likely to establish cooperative relationships, which improves resource exchange frequency and reduces risks related to supervision input and information leakage, thereby supporting Hypothesis 1. The estimated coefficient of Gwdegree is significantly positive (0.7867), indicating the presence of a core-periphery star structure in biotechnology cooperation networks. This suggests that organizations with more partners tend to converge within biotechnology cooperation networks, which promotes complex technology exchange and diffusion, reduces risks for new entrants, facilitates interorganizational information exchange, and ultimately enhances R&D efficiency. Therefore, Hypothesis 2 is supported.

With respect to exogenous attributes, the estimated parameter for organizational homogeneity is 0.0968, and its effect is not statistically significant. Organizations in biotechnology cooperation networks do not tend to cooperate with others of the same organizational type. This may be attributed to the increasing participation of organizations, which leads to heightened competition and a reduced willingness to cooperate with organizations of the same type. Consequently, Hypothesis 3 is rejected. The estimated coefficient of geographic homogeneity is significantly positive (2.6206), indicating a strong preference among biotechnology organizations for cooperation with others located in the same geographic region. A shorter geographical distance enhances communication and interaction among these organizations, which improves the efficiency of cooperation and facilitates the transfer and acquisition of tacit knowledge. This finding suggests that cross-regional cooperation has not yet become a widespread trend in current biotechnology cooperation networks, thereby validating Hypothesis 4.

The estimated coefficients of degree centrality and structural holes as control variables are significantly positive (0.0731, and 3.2341, respectively). This suggests that organizations are more inclined to cooperate with partners having higher degrees of centrality, and occupying structural holes within the networks. The estimated coefficient of stability (1.4867) demonstrates a significantly positive time dependence.

### 5.3 Robustness tests

**Figure 5:**
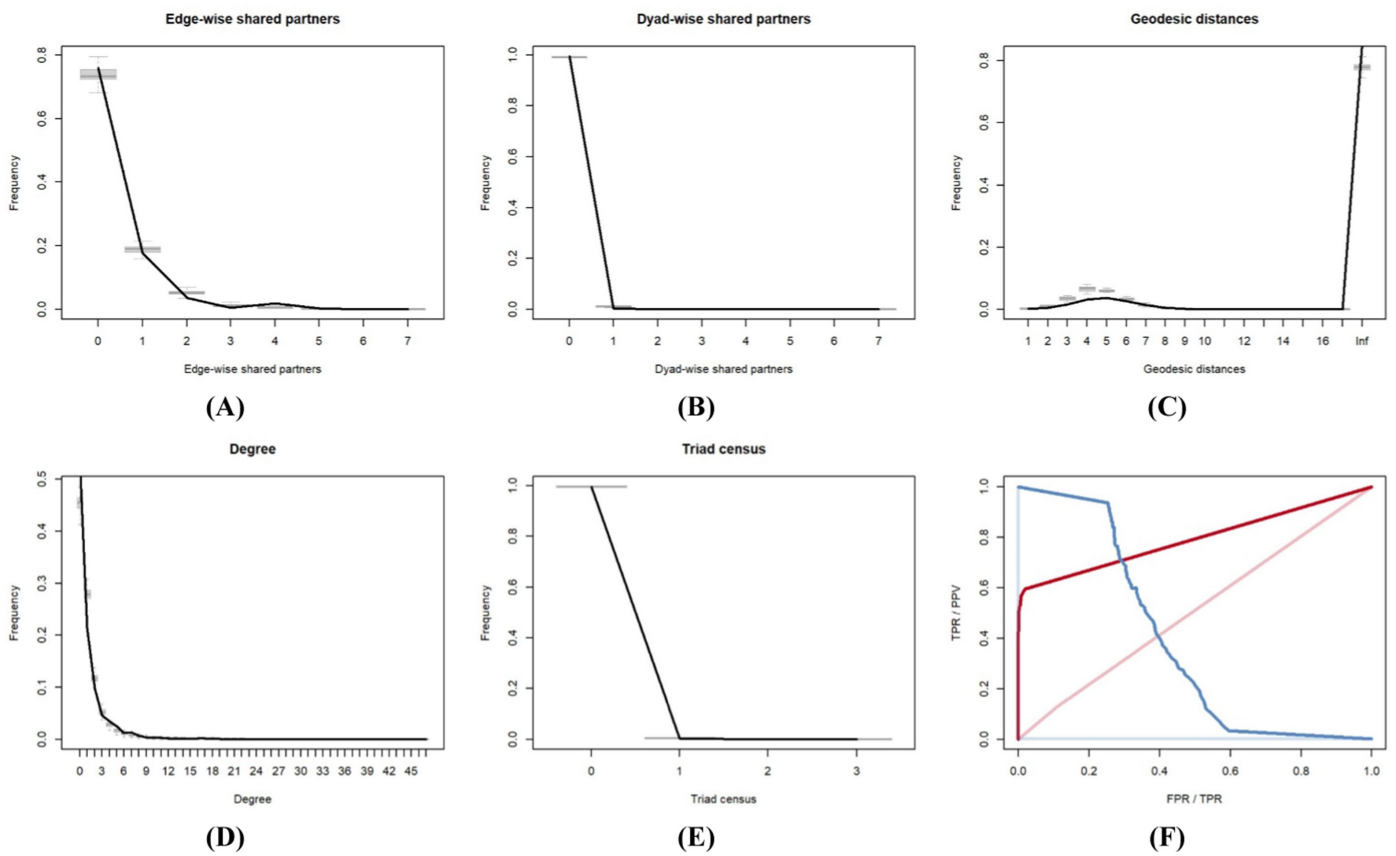
Goodness-of-fit assessment of the TERGM. **(A)** Goodness-of-fit based on edge-wise shared partners, **(B)** Goodness-of-fit based on dyad-wise shared partners, **(C)** Goodness-of-fit based on geodesic distances, **(D)** Goodness-of-fit based on degree, **(E)** Goodness-of-fit based on triad census, **(F)** Goodness-of-fit based on ROC and PR.

We referred to Leifeld et al. (22) and employed the goodness-of-fit (Gof) test to examine the robustness of the model fit. First, a series of simulated networks were generated using the observed values of the variables and the coefficient values derived from model estimation. A comparative analysis was subsequently conducted between the metrics obtained from these generated networks and those derived from actual observed networks. We chose edgewise shared partners, dyad-wise shared partners, geodesic distances, degrees, and triad censuses as the metrics for analysis. The results of these analyses are presented in Figures 5A–E. The black lines in the five subfigures indicate the distribution of each indicator in the actual network, and the gray lines and box plots represent the simulated network statistics. The reliability of the model estimation results increases when the distribution curve of the actual network aligns closely with the midpoint of the simulated network’s distribution interval. The observations in the initial five subplots in Figure 5 closely match the gray line, indicating that the TERGM developed in this study has a strong fit and robust estimation results.

The receiver operating characteristic (ROC) curve and the precision recall (PR) curve can also be employed to assess the Gof of the TERGM. Closer proximity of the ROC curve to the upper left corner indicates a superior fit of the model. The results of the ROC and PR curves in this study are shown in Figure 5F. Near the upper left corner of the figure is the ROC curve, and near the upper right corner is the PR curve. The ROC curve is prominently positioned in the upper left corner, indicating a superior fit between the constructed simulation network and the actual network. Furthermore, the estimation results of the TERGM are robust.

Second, a sensitivity analysis of the TERGM results is conducted by adjusting the decay parameter *α* of Gwesp and Gwdegree. Specifically, Model 1 presents the TERGM analysis results for the biotechnology cooperation network when the decay parameter is set to 0.2. Models 2, 3, and 4, respectively, represent the corresponding TERGM results when α is set to 0.4, 0.6, and 0.8. The detailed results are summarized in Table 6. Despite variations in the value of α , the significance levels and directions of the estimated parameters remain unchanged. These findings clearly demonstrate the remarkable robustness of the study’s findings.

**Table 6:** Sensitive analysis of the TERGM.

	Variables	Model 1 *α*=0.2	Model 2 *α*=0.4	Model 3 *α*=0.6	Model 4 *α*=0.8
Endogenous factors	Edges	−12.5422* [−13.5930; −9.3158]	−13.5092* [−14.7590; −10.2233]	−15.0009* [−16.2878; −12.5627]	−17.2697* [−18.7105; −13.4833]
	Gwesp	0.4365* [0.3595; 0.7764]	0.4278* [0.3480; 0.7872]	0.4227* [0.3305; 0.6905]	0.4205* [0.3393; 0.8223]
	Gwdegree	0.4155* [0.3818; 0.6475]	0.6381* [0.5889; 0.8744]	0.9692* [0.8998; 1.1666]	1.4750* [1.3783; 1.7586]
Exogenous factors	Organizational homogeneity	0.0919 [−0.0667; 0.2394]	0.0950 [−0.0386; 0.2435]	0.0989 [−0.0552; 0.2197]	0.1040 [−0.0086; 0.2021]
	Geographic homogeneity	2.6185* [2.4573; 2.7315]	2.6197* [2.4474; 2.7362]	2.6218* [2.4815; 2.7404]	2.6255* [2.5105; 2.7637]
Control variables	R&D capability	0.0001 [−0.0002; 0.0004]	0.0001 [−0.0003; 0.0004]	0.0001 [−0.0002; 0.0004]	0.0001 [−0.0003; 0.0004]
	Degree centrality	0.0785* [0.0360; 0.2926]	0.0750* [0.0293; 0.2947]	0.0713* [0.0311; 0.2235]	0.0692* [0.0329; 0.3172]
	Structural holes	2.6883* [0.8372; 3.1596]	3.0140* [1.1101; 3.5634]	3.5012* [2.0976; 4.0515]	4.2104* [1.9757; 4.8025]
Time-dependent effect	Stability	1.4864* [1.3810; 1.7592]	1.4866* [1.3794; 1.7617]	1.4869* [1.3996; 1.6851]	1.4878* [1.4158; 1.7679]

* 0 outside the confidence interval.

When analyzing time series data, the TERGM offers certain advantages and can partially mitigate endogeneity issues (111). By accounting for the dynamic changes in network, the TERGM can control for some confounding factors, thereby reducing the impact of endogeneity (112). However, the TERGM cannot eliminate endogeneity issues entirely (113). In contrast, the panel model can effectively control unobservable time-invariant factors at the observation level, thereby mitigating the impact of endogeneity (114). Therefore, to further enhance the reliability of the findings and to address potential endogeneity issues in the cooperation network, the panel logit regression model is introduced in the robustness test to examine the robustness of the conclusions for Hypotheses 1 and 2. The dependent variable is the presence of a network edge between node pairs. The independent variables include the presence of common partners between node pairs and the sum of their core-periphery degrees. Specifically, the presence of common partners between node pairs reflects the Gwesp of the TERGM, whereas the sum of their core-periphery degrees reflects the Gwdegree. Table 7 presents the estimation results of the panel logit regression model. When these results are compared with the TERGM results, the directions of the key variable coefficients are consistent. These findings indicate that the study’s conclusions remain valid after controlling for endogeneity, which significantly enhances the robustness of the findings.

**Table 7:** The panel logit regression model results.

Variable	Model 1	Model 2	Model 3
Common partner	1.9939*** (−0.1163)		1.8633*** (−0.1183)
Core peripheral		2.4110*** (−0.2345)	1.8191*** (−0.239)
Observations	5,912	5,912	5,912
Log likelihood	−1.94E+03	−2.05E+03	−1.91E+03

***, **, and * represent statistical significance at the 0.1, 1, and 5% levels, respectively; standard errors in parentheses.

## 6 Conclusions and implications

### 6.1 Main conclusions

This study analyzed the influences of endogenous and exogenous factors on the evolution of biotechnology cooperation networks by emphasizing the micromechanisms under these influences. Based on TERGM, we yielded the following conclusions.

First, time dependence is significant in the evolution of the biotechnology cooperation networks. The formation of cooperation is influenced by past interactions (41). On the one hand, long-term cooperations help organizations establish stable communication channels by reducing risks and uncertainties. On the other hand, previous interaction experiences affect organizations’ partners choosing and make them more inclined to select those with cooperation history (39). Second, both the transitivity and convergence positively affect the evolution of the biotechnology cooperation networks. This conclusion is consistent with Ma et al. (37) and Pan et al. (115). Transitivity enhances the efficiency of information diffusion, the accuracy of information acquisition, and the stability of networks. Convergence promotes organizations’ continuous attraction of external resources and enables complex technology exchange. Finally, geographical homogeneity has a significant positive effect on the evolution of biotechnology cooperation networks, which is consistent with the findings of Su et al. (45) and Teng et al. (85).

### 6.2 Policy implications

Drawing on our research findings, we present the following policy implications. Firstly, due to the time-dependent nature of the biotechnology cooperation networks, the formation of cooperative relationships is influenced by past interactions. Consequently, it is essential to consider policy lag effects (116). When formulating policies, governments should establish policy effect tracking and evaluating mechanism to assess policy outcomes regularly. Such comprehensive evaluations could be conducted every two or 3 years. Based on the evaluation results, policy directions should be properly adjusted to ensure adapted with the development of biotechnology industry (117, 118). Besides, it is necessary to engage experts in the development of industry evaluation index and to enhance the communication of policy adjustments (119).

Secondly, governments should address potential lock-in effects and obstacles that may arise from geographic homogeneity in cooperations. With unified technical standards, governments could establish innovation centers in key regions to promote cooperations across different regions (120). Besides, regional interest could be balanced by interregional coordination mechanism (121).

Thirdly, the biotechnology cooperation network exhibits endogenous structural tendencies of transitivity. Transitivity may lead to path-dependence and problems with local aggregation (69). As a result, local tightness and unbalanced cooperations may hinder the optimal resources allocation on whole level. Governments could establish information platforms to integrate information and facilitate communication among organizations (122). Additionally, governments could establish special support funds to provide financial incentives to encourage organizations to expand their cooperations. Besides, governments should strengthen policy publicity during implementation to reduce information asymmetry and policy resistance (123).

Finally, biotechnology cooperation networks also exhibit endogenous structural tendencies of convergence. Convergence can easily lead to information homogeneity (69). Government could establish special subsidies for small and medium enterprises to foster cooperations with diverse partners (124). Furthermore, government should formulate policies related to technology confidentiality and clarify the legal responsibilities associated with technology leakage. Besides, implement challenge should be considered such as uneven distribution of subsidy funds.

### 6.3 Limitations and opportunities

This study has several limitations that may offer opportunities for future research. Firstly, we mainly focused on the evolution of biotechnology cooperation networks in China. Although focusing solely on a single country is more suitable for proposing policy recommendations, it may also limit the generalizability of the findings. Further studies could investigate the evolution of biotechnology cooperation networks in other nations or explore international collaborations. Secondly, we only constructed cooperation networks based on patent data. Further studies may combine other forms of cooperations based on project or academic paper data to gain a comprehensive picture of biotechnology cooperation networks. Thirdly, future work could adopt machine learning models to enhance analytical capabilities. For instance, deep learning architectures like Graph Neural Networks (GNNs) could be employed to extract latent collaboration patterns from unstructured data (e.g., technical texts in patents or research abstracts), complementing traditional network analysis based on structured data. Finally, further studies could analysis the evolution of cooperation networks in other industries based on our methods and the micromechanisms framework.

## Data Availability

Publicly available datasets were analyzed in this study. This data can be found at: https://www.jianguoyun.com/p/DaYyclgQiM-aDRinlukFIAA.

## Footnotes

^1^ http://search.cnipr.com
